# Is the Other-Race Effect in Working Memory Due to Attentional Refreshing?

**DOI:** 10.5334/joc.263

**Published:** 2023-02-15

**Authors:** Philippe Schneider, Evie Vergauwe, Valerie Camos

**Affiliations:** 1 Université de Fribourg, Switzerland; 2 Université de Genève, Switzerland

**Keywords:** Other-Race Effect, Attentional Refreshing, Familiarity, Face Processing, Working Memory, Maintenance

## Abstract

The other-race effect is the observation that faces from another ethnicity induce worst recall performance than faces from one’s own ethnicity. This effect has been defined as a type of familiarity effect, with more familiar faces better recalled than less familiar faces. In this study, we tested the hypothesis that a working memory maintenance mechanism called attentional refreshing mediates the other-race effect and that faces from one’s own ethnicity are refreshed more efficiently than faces from other ethnicities. In two experiments, face ethnicity was orthogonally manipulated with cognitive load of a concurrent processing task in a complex-span paradigm (Exp. 1) and with the memory load in a Brown-Peterson paradigm (Exp. 2). Both cognitive and memory load effects are indices of the functioning of attentional refreshing. Testing Caucasian young adults, Caucasian and East-Asian faces were contrasted. Results from both experiments were congruent and against our initial hypothesis. The other-race effect in working memory does not appear to be supported by attentional refreshing. Furthermore, the results are congruent with the idea that faces as a whole are not maintained in working memory via attentional refreshing.

The familiarity of to-be-remembered material is known to impact performance in working memory (WM) tasks (Curby et al., 2009; Peeck & Zwarts, 1983). Among the different effects related to familiarity, the other-race effect (ORE) is considered as a kind of familiarity effect or perceptual expertise (Tanaka et al., 2013), because it relies on the occurrence of a type of faces encountered in day-to-day life (Stahl, Wiese, & Schweinberger, 2008; Stelter & Degner, 2018; Valentine & Endo, 1992). The ORE is the observation that faces from another ethnicity induce worse recall performances than faces from one’s own ethnicity in WM and long-term memory (LTM) settings (Rhodes et al., 2009; Tüttenberg & Wiese, 2022). Despite its importance in our daily life, the mechanism underlying the ORE in WM remains unknown. In the current study, we examined whether the ORE in WM tasks is due to a more efficient maintenance of own-race faces by attentional refreshing, relative to other-race faces.

Attentional refreshing is a maintenance mechanism that protects WM representations against decay and interferences by focusing central attention on them (Barrouillet et al., 2007; Barrouillet & Camos, 2021; Johnson, 1992; see Camos et al., 2018, for a review). It has been assumed that attentional refreshing relies in part on LTM, with WM representations being reconstructed based on information held in LTM (Barrouillet & Camos, 2015), akin to redintegration (Hulme et al., 1997). This would mean that information in WM that is also better represented in LTM would be refreshed more efficiently than information that is less well represented in LTM, because attentional refreshing could rely on this LTM information as support for reconstructing the WM representation. Such a mechanism could then explain the ORE, because own-race faces could rely more than other-race faces on LTM representations to withstand forgetting.

## The present study

The present study aimed at investigating whether the ORE is influenced by attentional refreshing, either by manipulating the opportunities to use refreshing and examining its impact on recall performance of faces (Experiment 1) or, conversely, by examining the impact of the number of to-be-memorized faces on the efficiency of refreshing (Experiment 2). In Experiment 1, refreshing opportunities were reduced by increasing the cognitive load (CL) of a concurrent task; CL referring to the amount of time during which a task occupies central attention divided by the total processing time allotted to the task (Barrouillet et al., 2007). In a complex-span task (i.e., a WM tasks in which item presentation is interspersed by a concurrent attention-demanding task), concurrent tasks with higher CL induce poorer recall performance than concurrent tasks with lower CL (Barrouillet, Bernardin & Camos, 2004), because higher CL tasks occupy central attention longer, and thus, less time can be dedicated to refreshing, compared to lower CL tasks. In Experiment 2, the memory load was manipulated in a Brown-Peterson task and its impact was measured on the reaction times (RTs) to the concurrent task. A Brown-Peterson task is akin to a complex span task, except that item presentation is grouped at the beginning of the trial, and the concurrent task is interspersed between the last memory item and the memory test. Because attentional refreshing is thought to be deployed in a sequential manner (i.e., representations being refreshed one after the other; Lemaire, Pageot, Plancher, & Portrat, 2018; Portrat & Lemaire, 2015), higher memory load should yield longer RTs to the concurrent task; the refreshing of the memory items postponing the processing of the distractors in the concurrent task (Camos et al., 2019; Vergauwe, Camos & Barrouillet, 2014).

**Figure 1:**
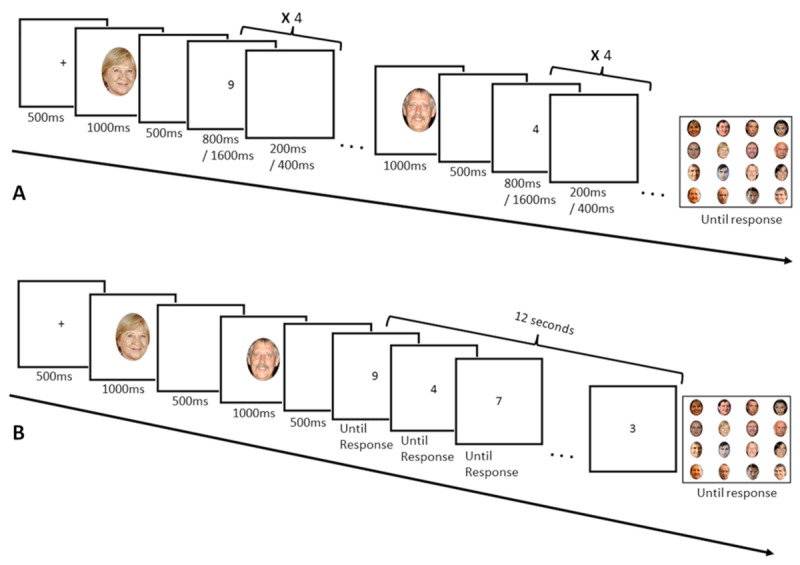
Schematic representation of a trial in Experiment 1 **(A)** and Experiment 2 **(B)**. Both examples show trials with a memory load of two items and Caucasian faces.

In Experiments 1 and 2, face ethnicity of the memoranda was orthogonally manipulated to the CL of a concurrent processing task in a complex-span paradigm (Figure 1A) and to the memory load in a Brown-Peterson paradigm (Figure 1B), respectively, using a parity judgment task as concurrent task in both. Raw data can be found on the following OSF page: https://osf.io/n9zsg/.

## Experiment 1

Experiment 1 investigated how face ethnicity influences recall performance in a complex span paradigm, in which the CL of the concurrent task was manipulated. We expected that faces from participants’ own ethnicity should benefit more from the refreshing opportunities in the low CL condition compared to the high CL condition, if own-ethnicity faces are refreshed more efficiently than other-ethnicity faces. This would lead to an interaction between CL and face ethnicity manipulations.

### Methodology

#### Participants

Forty students from the University of Geneva (3 males, mean age: 20 ± 1.7 years) participated in this experiment for partial course credits. All signed an informed consent form. This study received ethical approval by IRB of the University of Geneva. To assure that our participants had low familiarity with Asian faces, we asked them in a post-experiment question whether they have lived for more than 3 months (cumulatively) in an East-Asian country (Korea, Japan, China, etc.). Answering yes to this post-experiment question led to discard their data from the analysis. We assumed that people that lived for more than 3 months in east-Asia would be more accustomed to Asian faces, and thus would not incur the ORE reliably. The sample size was determined on the basis of former experiments which reliably showed CL effects with a similar sample size (Camos et al., 2019).

#### Material

The faces used as memoranda were selected from the *10k US Adults Faces* database (Bainbridge, Isola, & Oliva, 2013). In this database, all faces were rated on their attractiveness, age (on a 5 points Likert scale) and sex. In addition, the authors also evaluated the intrinsic memorability of each face via a continuous recognition task. Series of faces were presented, and participants had to decide whether each face had already been presented. The hit-rate and false alarm rate for each face was recorded. Sixty-three Asian faces were randomly selected from the database. Approximately half of those faces were from women. Each of them was paired with a Caucasian face that had similar scores on attractiveness, age, sex, hit-rate and false alarm rate (Table 1).

**Table 1:** Mean attractiveness (scale from 1, not at all, to 5, very much), age (scale from 1, very young, to 5, very old), and hit and false alarm rates for both Asian and Caucasian faces (SD in brackets).


	ATTRACTIVENESS	AGE	HIT RATE	FALSE ALARM

Asian Faces	2.89 (0.75)	2.48 (0.80)	0.61 (0.10)	0.12 (0.08)

Caucasian Faces	2.88 (0.81)	2.50 (0.78)	0.61 (0.10)	0.12 (0.07)


#### Procedure

The experiment was divided in two blocks, one for each kind of faces. The blocks order was counterbalanced across participants. Each block used an increasing length procedure, in which the number of to-be-maintained faces increased gradually from one to six. There were six trials for each list length, three in each CL condition, totalling 36 trials per block. All blocks started with length-1 trials. The block stopped if no trial from a given length was successful, or after participants finished length-6 trials. The order in which high and low CL trials were presented was randomized for each list length.

The concurrent task was a parity judgment task, in which participants judged the parity of each digit presented one after the other on screen. Participants had to press the right or left mouse button for even or odd digits, respectively. Four digits (1 to 9, 5 excluded) were presented sequentially on screen. Each digit stayed on screen for either 800 ms followed by a 200 ms ISI, or 1600 ms followed by a 400 ms ISI, in the high and low CL conditions, respectively. Digits were selected randomly.

The experiment started with a training of the secondary task. Twenty-four digits appeared sequentially on screen (12 for each CL condition). Next, there were four training trials, two with 2 faces and two with 3 faces. Trainings used both kind of faces alternatively. Faces used in the trainings were not used in the experimental trials.

Each trial started with a screen informing of the CL condition (High or Low). Participants had to press the space bar to start the trial. When they pressed it, they had to start repeating “BA-BI-BU” out loud to minimize verbal recoding and the use of articulatory rehearsal. A fixation cross appeared in the centre of the screen, followed by the first face to remember. After the face disappeared, the concurrent task started. This would repeat for the length of the trial. After the parity phase following the last-presented face, participants were presented with an array of sixteen faces and had to click on the presented faces in their order of presentation using the computer mouse. Participants could not correct themselves and stopped repeating “BA-BI-BU” as soon as the recognition phase started. After the participant had responded, the information screen for the next trial was prompted. Presentation timings are shown in Figure 1A.

Within each block, each face was used twice as memorandum, once per CL condition. The faces presented during response phase were intermixed with non-target faces of the same race. Each face was used a maximum of 5 times as non-target.

### Results

**Figure 2:**
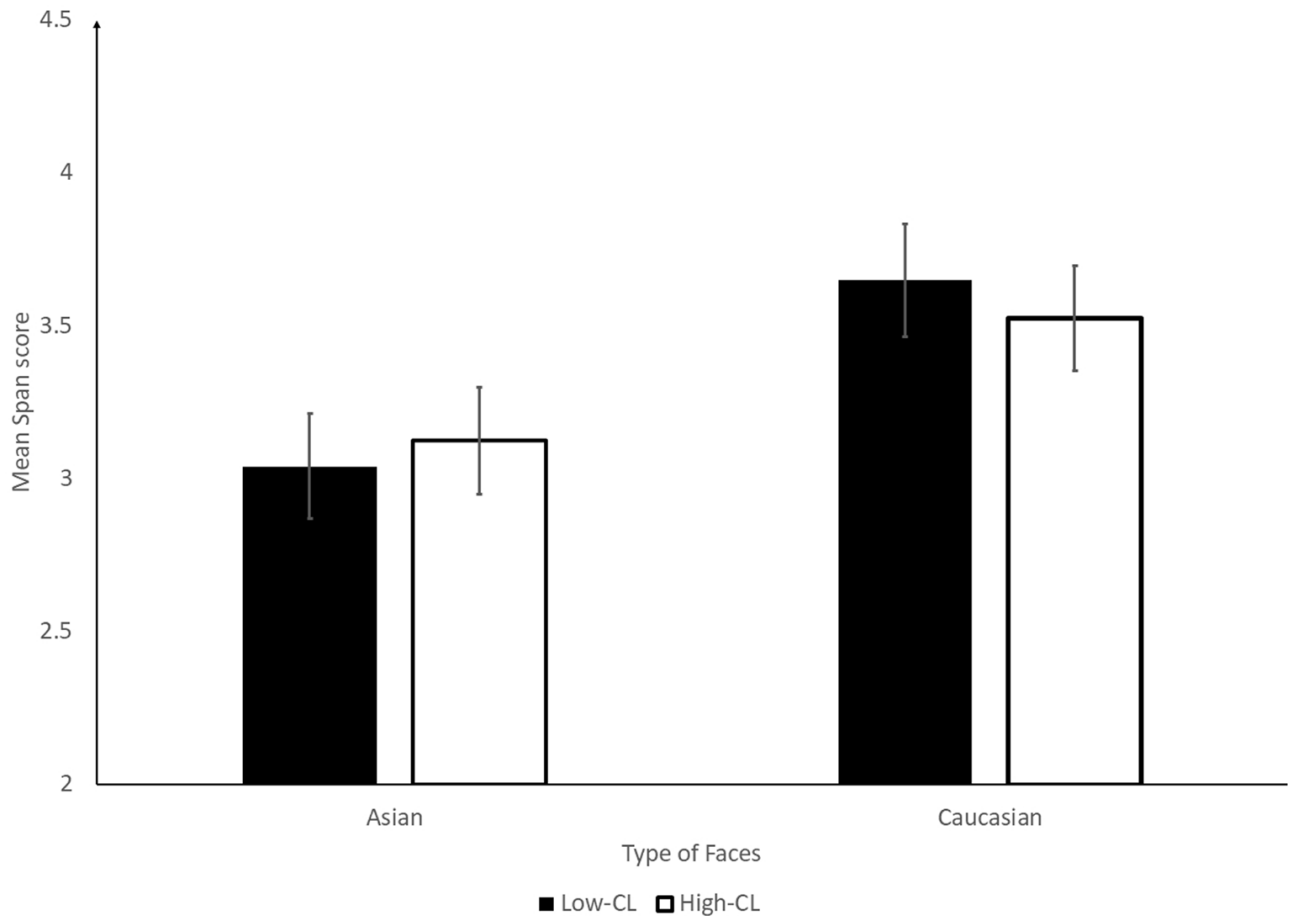
Mean span score as a function of cognitive load (CL) of the concurrent task and type of faces in Experiment 1. Error bars are standard errors.

No data were excluded on the basis of the post-experiment question. Moreover, all participants reached at least 70% of correct responses to the parity judgement task. This was our cut-off for excluding participants to ensure task rules’ compliance (see Camos et al., 2019; Vergauwe et al., 2014, for more details). Recognition performance was scored as a span (Conway et al., 2005). Each successful trial (i.e., all faces recognized in the correct presentation position) was worth one point, then we summed the points for each experimental cell and divided it by three (total number of trials per experimental cell). Span scores were analysed in a 2 (CL: high or low) × 2 (face type: Asian or Caucasian) repeated measure Bayesian ANOVA with the BayesFactor Package (Morey & Rouder, 2018) in R (R Core Team, 2019). The best model included only the main effect of face type, BF_10_ = 2.8 × 10^3^, with Caucasian faces yielding better performance than Asian faces (Mean span = 3.6 ± 1.1 and 3.1 ± 1.1 for Caucasian and Asian faces, respectively; Figure 2). However, evidence was gathered against an effect of CL on recall performance, BF_exclusion_ = 5.8 (Mean span = 3.3 ± 1.1 and 3.3 ± 1.2 for fast and slow pace, respectively). In addition, there was weak evidence against the presence of the interaction, BF_exclusion_ = 2.5.

### Discussion

Caucasian faces led to better recognition performance compared to Asian faces. Because we ensured that our participants were more familiar with Caucasian faces than with East-Asian faces, we are confident that this finding evidenced the presence of an ORE in our data. Contrary to our expectations, we found evidence against the effect of CL. If this absence of effect is confirmed, faces could be added to the list of visuospatial materials that proved to be immune to CL manipulations (Ricker & Cowan, 2010; Ricker & Vergauwe, 2020; Vergauwe, Camos & Barrouillet, 2014). Finally, we found weak evidence against the interaction between CL and face ethnicity, going against our hypothesis.

## Experiment 2

Experiment 2 implemented a Brown-Peterson task, in which the impact of memory load and face ethnicity on reaction time to the concurrent task was assessed. Since attentional refreshing postpones the processing of a concurrent task, a higher number of images to maintain should postpone the processing of the concurrent task (Vergauwe et al. 2014). In addition, if own-race faces are refreshed more efficiently (i.e., more quickly) than other-race faces, then the concurrent task should be less postponed by own-race faces than by other-race faces. Thus, the memory load effect on RTs to the concurrent task should be smaller for own-race compared to other-race faces, leading to an interaction between memory load and face ethnicity.

### Methodology

#### Participants

Thirty-two students from the University of Geneva (8 men, mean age = 20.5 ± 2.2 years) participated in this experiment for partial course credits. None of them participated in the previous experiment and they had to respond to the same post-experiment question as in Experiment 1 for the same reason. All signed an informed consent form. This study received ethical approval by IRB of the University of Geneva. The sample size was determined on the basis of former studies which showed a reliable effect of memory load on concurrent task RTs (Camos et al., 2019).

#### Procedure

This experiment followed the same procedure as Experiment 1 with a few differences. The faces were presented one after the other at the beginning of a trial, with the concurrent task happening after all images are presented and before the recognition phase. The concurrent task lasted for a fixed 12-s. duration. Participants had to sort as many digits as possible. Digits changed as soon as participants answered. The experiment was blocked as in Experiment 1 and followed the same increasing length procedure, starting with length-1 and with a maximum of length-6. There were 6 trials per list-length and type of face. Before the experimental trials, participants had a training for the parity phase containing 20 digits. There were 4 training trials to the Brown-Peterson task, alternating with Caucasian and Asian faces.

### Results

The data of one participant was excluded because they answered “yes” to the post-experiment question. No participant had less than 70% of correct responses to the parity judgement task. As in Vergauwe et al. (2014), we only kept the data of participants who had more than 50% of correct trials (all faces recalled in the correct position) to ensure they followed instructions. Three participants were excluded on this basis, leaving us with a final sample of 28 participants for the analysis.

We computed the span score for each participant and each type of faces. We then used the span score as dependent variable in a paired Bayesian t-test with faces (Asian or Caucasian) as independent variable. Results showed that Caucasian faces induced better recognition performance than Asian faces, BF_10_ = 991 (Mean span = 4.1 ± 0.7 and 3.6 ± 0.7 for Caucasian and Asian faces, respectively).

**Figure 3:**
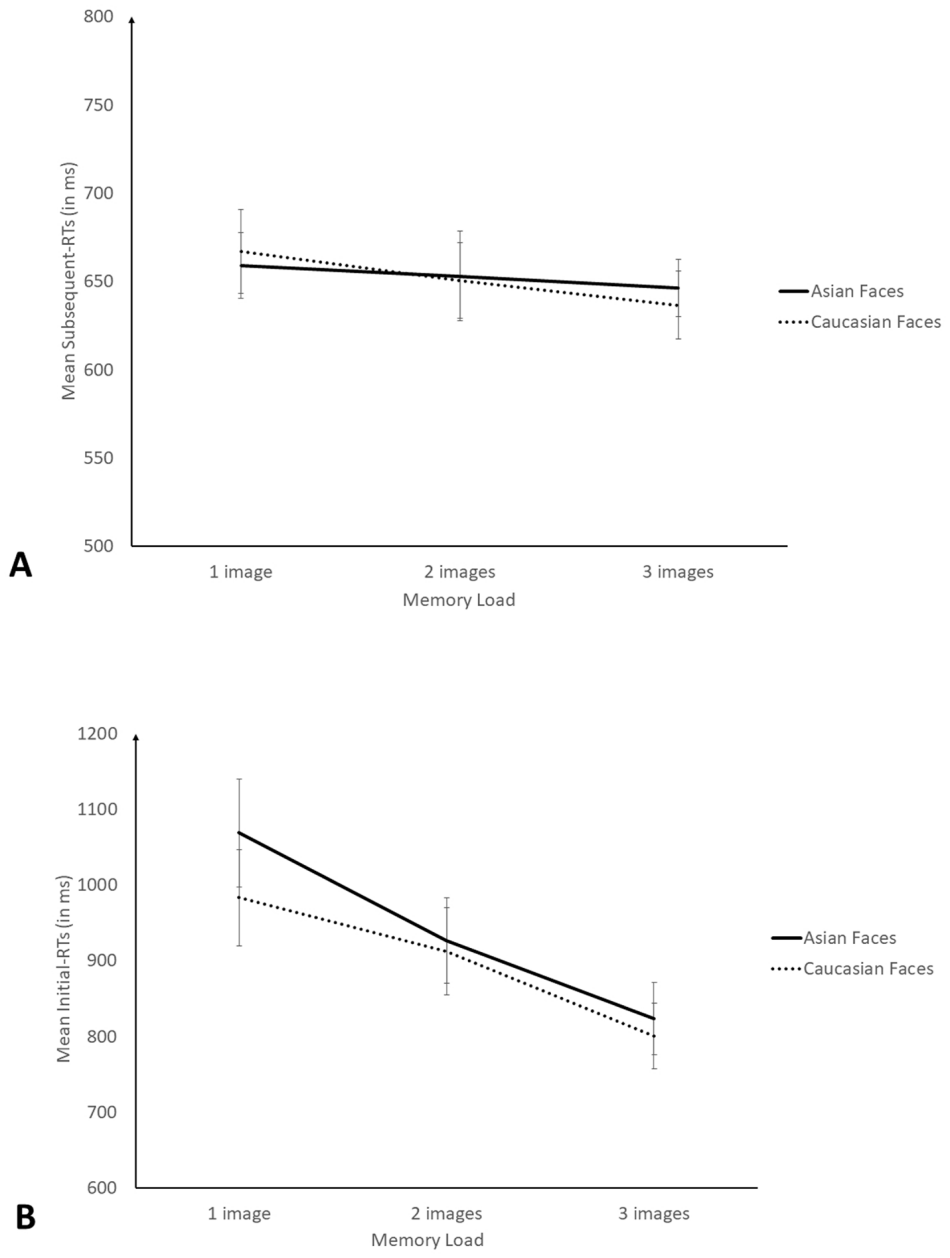
**A:** Mean Subsequent RTs (in ms) as a function of memory load and type of faces and. **B:** Mean Initial RTs (in ms) as a function of memory load and type of faces in Experiment 2. Error bars are standard errors.

We then analysed the RTs to the parity judgement task as in previous studies (Camos et al., 2019; Vergauwe, et al., 2014). We separated the RTs to the first digit of the parity phase (hereafter: Initial-RTs) from the mean RTs to the other digits (digits in position 2 and further, hereafter: Subsequent-RTs). Indeed, it has been shown that Initial-RTs were always longer than Subsequent-RTs from the same processing phase, and thus should be analysed separately because they depend on different cognitive processes, Initial-RTs indexing memory consolidation while Subsequent-RTs assess refreshing (Engle et al., 1992; Friedman & Miyake, 2004; Jarrold et al., 2011). In addition, memory load conditions (i.e., trials of a particular list length) were included in the analysis only if more than 2/3 of participants succeeded perfectly to the memory task (i.e., all faces recalled in the correct position) in 2 or more trials from the memory load condition in question. Hence, trials with memory load of 4 and further were discarded on this basis.^1^ We then computed the mean Subsequent-RTs as a function of memory load and type of faces and used it as a dependent variable in a 2 (Type of face: Asian or Caucasian) × 3 (memory load: 1 to 3) repeated measure Bayesian ANOVA. There was evidence against the inclusion of the type of faces, BF_exclusion_ = 6.1 (Mean Subsequent-RTs Caucasian face: 651 ms ± 113 ms; Subsequent-RTs Asian face = 652 ms ± 107 ms), against an effect of memory load, BF_exclusion_ = 5.0 (Mean Subsequent-RTs 1 image: 663 ms ± 111 ms; Mean Subsequent-RTs 2 images: 652 ms ± 124 ms; Mean Subsequent-RTs 3 images: 642 ms ± 94 ms) and against the interaction, BF_exclusion_ = 7.7 (Figure 3A).

Next, we analysed the Initial-RTs, as for the Subsequent-RTs, which led to keep trials in which participants succeeded in the memory task and a memory load from 1 to 3. We then computed mean Initial-RTs as a function of memory load and face type for all participants. Using this score as a dependent variable in a 2 (type of faces: Asian or Caucasian) × 3 (memory load: 1 to 3) Bayesian repeated measure ANOVA showed that Initial-RTs were impacted by the memory load only, BF_10_ = 2.7 × 10^4^ (Figure 3B). We found evidence that smaller memory load induced longer Initial-RTs compared to higher memory load, BF_inclusion_ = 3.3 × 10^4^ (Mean Initial-RTs 1 image: 1’027 ms ± 357 ms; Mean Initial-RTs 2 images: 920 ms ± 299 ms; Mean Initial-RTs 3 images: 812 ms ± 239 ms). We found weak evidence against an effect of type of faces, BF_exclusion_ = 2.5 (Mean Initial-RTs Asian face: 964 ms ± 346 ms; Mean Initial-RTs Caucasian face: 913 ms ± 316 ms), and substantial evidence against the interaction, BF_exclusion_ = 6.0.

### Discussion

Experiment 2 replicated the ORE effect; faces from participants’ own ethnicity were better recalled than faces from another ethnicity. Regarding the Subsequent-RTs, the memory load and type of faces had no clear effect. Since the effect of memory load on Subsequent RTs indexes the functioning of attentional refreshing (Camos et al., 2019; Vergauwe, et al., 2014), our data goes against the hypothesis that own-race faces are refreshed more efficiently than other-race faces. Finally, Initial-RTs were influenced only by the memory load, but in a rather unexpected way as higher memory load induced shorter Initial-RTs. This trend is in the opposite direction to what was reported with other types of memory material. Although this could pinpoint a specificity of face processing, it could be due by the fact that we used an increasing length procedure. Trials with more memoranda were always at the end of a block, i.e., when participants benefited from the most amount of training to the task.

## General discussion

In two experiments, we provided further evidence for the impact of ORE in WM, with better performance with faces from participant’s ethnicity than faces from another ethnicity. Hypothesizing that ORE in WM would result from the use of attentional refreshing, we gathered evidence against this hypothesis. More specifically, there was evidence against an effect of CL on memory performance in Experiment 1, and against an effect of memory load on Subsequent-RTs to the concurrent task in Experiment 2, both effects being indexes of attentional refreshing. Taken together, these results are congruent with the idea that faces were not maintained via attentional refreshing.

The difference between recall performance of other-race and own-race faces could thus be due to other mechanisms. For example, Tüttenberg & Wiese (2022) showed a widespread modulation of event-related potentials between own-race and other-race, both at perceptual and mnemonic levels. Thus, differences in recall performance between both type of faces could be due to differences in how we perceive faces, with an advantage to own-race faces. These differences could then be reflected in later processing stages such as encoding in WM and in LTM, hence and affecting memory performance.

Although we replicated the other-race effect in WM, the difference in memory performance between Caucasian and Asian faces in our study could be partly driven by qualitative differences between the two sets of faces. Although both sets of faces were equated on several variables, known for influencing their memorability (age, sex, attractiveness), Caucasian faces have more intrinsic differences between them than Asian faces (for example, a larger variability of hair colour). This would make the Caucasian faces more easily distinguishable from each another, and thus elevates their probability to be correctly recognized. Future research on ORE should aim to take this variability issue into account very carefully.

To conclude regarding attentional refreshing, previous studies have reported that some materials were not influenced by CL manipulations. They were always in the visual domain, but concern mainly uncommon memoranda (e.g., fonts, unconventional characters, positions around a circle; Ricker & Cowan, 2010; Ricker & Vergauwe, 2020; Vergauwe, et al., 2014). This has led researchers to suggest that, to be unrefreshable, memoranda should have little or no prior representation in LTM (Ricker & Vergauwe, 2020). However, since faces are ubiquitous in day-to-day lives, they cannot be considered as uncommon. It is then difficult to argue that faces, especially those from our own ethnicity, do not benefit from any LTM representations. Thus, LTM representation does not seem to be the main factor influencing the refreshability of information in WM. Alternatively, it is possible that our observations are due to the fact that memory performance was assessed in a recognition test. It has been shown that recognition of visuo-spatial information may not require any active maintenance in WM to be performed correctly (Uittenhove, Chaabi, Camos, & Barrouillet, 2019). Congruently, our participants may have been able to recognize faces without having to actively refresh them, but rather based on some episodic LTM traces. Future research should aim at investigating this hypothesis to understand what contributes to the ORE in WM.

## Data Accessibility Statement

This work was supported by the Swiss National Science Foundation by grant 100019_175960 to Valérie Camos. Data are available on OSF https://osf.io/n9zsg/.

## Footnotes

Twenty-five participants succeeded in two or more trials with a list-length of 3; Fourteen succeed in 2 or more trials with list length of 4; Five participants succeeded in two or more trials with a list length of 5; 2 participants succeeded in in 2 or more trials with a list length of 6.
